# Case Series about the Changed Antiplatelet Protocol for Carotid Endarterectomy in a Teaching Hospital: More Patients with Complications?

**DOI:** 10.1055/s-0038-1675566

**Published:** 2018-11-05

**Authors:** Martijn S. Marsman, Denise M.D. Özdemir- van Brunschot, Abdelkarime Kh Jahrome, Nic J.G.M. Veeger, Wouter J. Schuiling, Frank G. van Rooij, Giel G. Koning

**Affiliations:** 1 Department of Vascular Surgery, Medical Center Leeuwarden, Leeuwarden, the Netherlands on behalf of HeelkundeFriesland.nl; 2 Department of Vascular Surgery, Augusta Krankenhaus, Düsseldorf, Germany; 3 Department of Epidemiology, MCL Academy, Medical Center Leeuwarden, Leeuwarden, the Netherlands; 4 Department of Neurology, Medical Center Leeuwarden, Leeuwarden, the Netherlands

**Keywords:** acetylsalicylic acid, ASA, bleeding, carotid endarterectomy, carotid artery, clopidogrel, dipyridamole, complication

## Abstract

**Introduction** In the Netherlands, clopidogrel monotherapy increasingly replaces acetylsalicylic acid and extended release dipyridamole as the first-choice antiplatelet therapy after ischemic stroke. It is unknown whether the risk of peri- and postoperative hemorrhage in carotid artery surgery is higher in patients using clopidogrel monotherapy compared with acetylsalicylic acid and extended release dipyridamole. We therefore retrospectively compared occurrence of perioperative major and (clinical relevant) minor bleedings during and after carotid endarterectomy of two groups using different types of platelet aggregation inhibition after changing our daily practice protocol in our center.

**Material and Methods** A consecutive series of the most recent 80 carotid endarterectomy patients (November 2015–August 2017) treated with the new regime (clopidogrel monotherapy) were compared with the last 80 (January 2012–November 2015) consecutive patients treated according to the old protocol (acetylsalicylic acid and dipyridamole). The primary endpoint was any major bleeding during surgery or in the first 24 to 72 hours postoperatively. Secondary outcomes within 30 days after surgery included minor (re)bleeding postoperative stroke with persistent or transient neurological deficit, persisting or transient neuropraxia, asymptomatic restenosis or occlusion, (transient) headache. Reporting of this study is in line with the ‘Strengthening the Reporting of Observational Studies in Epidemiology’ statement.

**Results** Although statistical differences were observed, from a clinical perspective both patients groups were comparable. Postoperative hemorrhage requiring reexploration for hemostasis occurred in none of the 80 patients in the group of the clopidogrel monotherapy (new protocol) and it occurred in one of the 80 patients (1%) who was using acetylsalicylic acid and dipyridamole (old protocol). In three patients (4%) in the clopidogrel monotherapy and one patient (1%) in the acetylsalicylic acid and extended release dipyridamole protocol an ipsilateral stroke was diagnosed.

**Conclusion** In this retrospective consecutive series the incidence of postoperative ischemic complications and perioperative hemorrhage after carotid endarterectomy (CEA) seemed to be comparable in patients using clopidogrel monotherapy versus acetylsalicylic acid and extended release dipyridamole for secondary prevention after a cerebrovascular event. This study fuels the hypothesis that short- and midterm complications of clopidogrel and the combination acetylsalicylic acid and extended release dipyridamole are comparable.

The neurological and vascular surgical communities established guidelines for antiplatelet therapy as secondary prevention after ischemic stroke or transient ischemic attack (TIA). 1 The American Heart Association guideline states that the following four antiplatelet regimes are equivalent in the reduction of the risk of ischemic stroke 2 :

- Acetylsalicylic acid (ASA)
- Clopidogrel
- Ticlopidine
- Combination of ASA and dipyridamole (ASA-D)

Platelet aggregation inhibition is an essential part of secondary prevention after TIA or ischemic stroke. International guidelines approve different antithrombotic regimens for this induction. 2 3 Based on the ESPRIT trial (Level of Evidence 1d [LoE 4 ]) the initial antiplatelet regime after TIA or ischemic stroke of presumed arterial origin consisted of ASA 80 mg once a day and extended release dipyridamole 200 mg twice a day. 3 In the PRoFESS (Prevention Regimen for Effectively Avoiding Second Strokes) trial no differences in cognitive decline or recurrent stroke were found when comparing ASA 25 mg tablets in combination with extended release dipyridamole 200 mg (ASA-D) twice a day versus clopidogrel 75 mg once a day. 5

With the PRoFESS trial in mind the antithrombotic treatment protocol in the Medical Center Leeuwarden (MCL) was changed from ASA-D to clopidogrel monotherapy in November 2015. Contributory reasons for changing this protocol were the lack of difference in outcome for secondary prevention of a ischemic event between clopidogrel and ASA-D, an increased incidence of headache in patients after starting dipyridamole, a potentially higher compliance for monotherapy, and a cost reduction as clopidogrel monotherapy seems to be less costly then ASA-D. 6 7 Knowledge about the effectiveness, complications, and costs of clopidogrel monotherapy versus ASA-D may provide insights for other paying parties, such as health insurance companies and hospitals.

Besides lifestyle changes and medication to prevent recurrent TIA or ischemic stroke in the future, patients with a significant and symptomatic carotid artery stenosis require a carotid endarterectomy (CEA). Although antiplatelet medication is important as secondary prevention, it also increases the risk of perioperative hemorrhage. 8 9 To date, it is unknown whether the risk of perioperative hemorrhage is higher in patients using clopidogrel monotherapy compared with those using ASA-D when undergoing CEA. ASA and clopidogrel monotherapy taken 24 hours before CEA increased post- or perioperative (cervical) bleeding risks. 8 10 Knowledge of the risk of perioperative hemorrhage in clopidogrel versus ASA-D is important because this can cause a potentially life-threatening situation. In this study, differences in perioperative complications associated with clopidogrel monotherapy versus ASA-D were evaluated in patients who underwent CEA for symptomatic internal carotid artery stenosis. The first aim was to scientifically evaluate outcomes of this safety analysis to provide insight in the complication rate of the operative carotid population of the MCL. These first results on this subject could be used as step up data for a randomized clinical trial.

## Material and Methods

### Patients

Between the change in protocol (November 1, 2015) and the moment of analysis a consecutive series of 80 patients who were treated with clopidogrel monotherapy (November 1, 2015–August 31, 2017) were compared with the last 80 consecutive patients treated according with ASA-D (January 1, 2012–November 1, 2015). Antiplatelet therapy was peri- and postoperatively continued in both groups. The perioperative period is defined as the duration between the event (stroke or TIA) after which the antiplatelet therapy started and the moment of the CEA. Patient data were retrieved from the electronic patient files and were analyzed retrospectively. Patients were all invited at the outpatient department for follow up physical examination and carotid ultrasound.

- Inclusion criteria:
  - Undergoing unilateral CEA for a symptomatic internal carotid artery stenosis (> 50% for the male and > 70% for the female patients). 11
  - Every patient who underwent a primary CEA in the period from the January 1, 2012 until August 31, 2017 at the MCL.
- Exclusion criteria:
  - Use of vitamin K or factor X-a inhibitors.
  - Use of other platelet inhibitor combinations.
  - Combination of more surgical interventions at the same time.

### Preoperative Procedure

As part of standard evaluation all patients with TIA or ischemic stroke as diagnosed by an experienced vascular neurologist underwent either computed tomographic angiography or magnetic resonance angiography of the cerebropetal arteries in combination with carotid ultrasonography. When patients were diagnosed with a symptomatic ipsilateral carotid stenosis (≥ 50% in male patients and ≥70% in female patients) they were referred to the vascular surgeon. This is according to the Dutch guidelines that also recommends to perform a CEA within 14 days after the initial ischemic event. 11 12 Next to these criteria, when the patient is scheduled for CEA in our center, the patient is referred to a dedicated and experienced carotid-team, consisting of a vascular surgeon, neurologist, anesthetist, vascular internist, and a specialized nurse for cardiovascular screening.

### Surgical Procedure

Surgical procedures were unchanged during the study period. Conventional CEA was performed under either general anesthesia or plexus anesthesia. 13 Conventional CEA was performed, as the carotid eversion technique is not used in MCL given the associated decreased baroreceptor sensitivity which may be related to difficult postoperative tension regulation, and because of the damage that can occur from cutting through the carotid plexus. 14 15 A 5,000 international units of heparin were given intravenously during CEA. No other hemostatic agents were used. A wound drain was not placed routinely, when placed, it could be removed when it produced less than 25 mL within 24 hours. The use of a carotid patch angioplasty is not unequivocally proven. 16 However, in the Dutch vascular surgery guidelines it is recommended. 17 At the MCL the use of a carotid patch angioplasty depends on the diameter of the carotid artery as can be assessed intraoperatively. All operations were performed by experienced vascular surgeons, all of whom had performed more than 200 CEA procedures. In accordance with our CEA protocol, all patients were closely monitored for the first 24 hours consisting of vital signs, repeated neurological tests and wound inspection. 18

### Endpoints

The primary endpoint was any bleeding (within 30 days after surgery) requiring hemostasis at the operation room. Secondary outcomes were death within 30 days after surgery, minor bleeding, rebleeding, postoperative ipsi- or contralateral stroke, or TIA, cranial nerve neuropraxia, restenosis or reocclusion, and headache (transient or persisting). Neuropraxia symptoms of cranial nerves were diagnosed by an experienced consultant vascular neurologist.

**Fig. 1:**
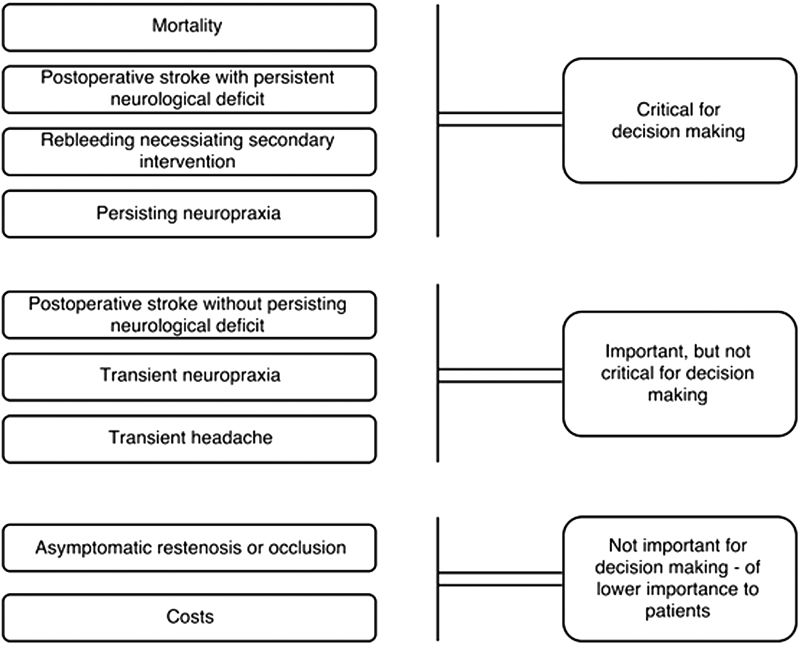
Example of hierarchy of outcomes according to the GRADE classification, 4 19 adapted for CEA patients.

Endpoints were graded ( Fig. 1 ) according to importance for patients according to the GRADE Working Group (Grading of Recommendations Assessment, Development and Evaluation). 19 This study was conducted and reported in line with the ‘Strengthening the Reporting of Observational Studies in Epidemiology’ statement (STROBE). 20

### Statistics

All statistical analyses were performed using SPSS version 24.0 (IBM Corp. Armonk, NY). Values were depicted as mean with standard deviation and number of cases with percentages. Group differences at baseline were assessed using Students *t* -test or Mann–Whitney *U* -test for continuous data (depending on normality of data) and Fisher's exact test or Pearson's Chi-square test for categorical data.

Fisher's exact test was used to test for differences in the occurrence of primary endpoint events. In addition, the exact 95% confidence intervals (CIs) of point estimates were calculated using the Clopper–Pearson method. For all secondary outcomes, the same analysis approach was used. A *p* -value of less than 0.05 was used for all tests to indicate statistical significance.

## Results

Between January 2012 and August 2017, a total of 4,514 patients with a stroke or TIA were treated in the MCL. Of these 4,514 patients 252 underwent CEA in line with the indications stated in international carotid guidelines. After exclusion of 92 patients because of predefined criteria, 80 consecutive patients receiving clopidogrel monotherapy were included. In addition, the most recent 80 consecutive patients that used ASA-D were included.

Overall, 73% of the 160 CEA were performed within 14 days after the initial ischemic event. In cases where the time frame of treatment was not attained, the reason for this was patient delay or prehospital referral delay. Patient demographics are shown in Table 1 and perioperative parameters are presented in Table 2 .

**Table 1:** Patients demographics and surgical parameters, stratified by antiplatelet therapy regime

	Clopidogrel ( *n* = 80)	ASA-D ( *n* = 80)	*p* -Value
Period	November 2015–August 2017	January 2012–November 2015	
Mean age in y (SD)	73 (9.8)	71 (10.8)	0.19
Male	46 (58)	55 (69)	0.19
Comorbidity a	59 (74)	72 (90)	0.01
Hypertension	45 (47)	58 (42)	
Diabetes	20 (21)	23 (17)	
Hypercholesterolemia	31 (32)	57 (41)	
Smoking	53 (66)	49 (61)	0.49
Presenting event			0.87
TIA	36 (45)	38 (47)	
Ischemic stroke	44 (55)	42 (53)	
Recurrent events between first presentation and CEA	0 (0)	0 (0)	NC

Abbreviations: ASA-D, acetylsalicylic acid and extended release dipyridamole; CEA, carotid endarterectomy; NC, not computable; SD, standard deviation; TIA, transient ischemic attack.

*Note* : Data are presented as *n* (%) unless stated otherwise.

a one or more.

**Table 2:** Perioperative parameters

	Clopidogrel ( *n* = 80)	ASA-D ( *n* = 80)	*p-* Value
Right sided CEA	34 (43%)	35 (44%)	1.00
Median delay from event to surgery in d (IQR)	11 (8–14)	11 (9–14)	0.96
Patch used	39 (49)	70 (88)	< 0.001
Dacron	2 (5)	1 (1)	
Biopatch	36 (92)	67 (96)	
Venous	1 (3)	2 (3)	
Shunting needed	20 (25)	18 (23)	0.85
Median duration of surgery in min (IQR)	90 (75–100)	90 (90–100)	0.08
Anesthesia			0.50
General	78 (98)	80 (100)	
Plexus	2 (2)	0 (0)	

Abbreviations: ASA-D, acetylsalicylic acid and extended release dipyridamole; Biopatch, bovine; IQR, Interquartile Range.

*Note* : Data are presented as *n* (%) unless stated otherwise.

Although significant differences were found in Tables 1 and 2 , these differences were considered to be not clinically relevant.

Although significant differences were found in Table 2 , these differences were considered not to be clinically relevant.

Complications are shown in Table 3 . Postoperative hemorrhage requiring return to the operation room occurred in no patients in the clopidogrel monotherapy protocol and in one (1%) in the ASA-D protocol. Three patients using clopidogrel and one patient using ASA-D developed an ipsilateral ischemic stroke. In the 30 day follow-up period two patients died, both in the clopidogrel group. One patient died because of a postoperative ipsilateral stroke and one due to an aspiration pneumonia. The CT-scan (computed tomography scan) of the patient with the stroke showed no recent ischemia or bleeding in the brain. There was, in contrast to a CT prior to the CEA, no significant stenosis of the internal carotid artery. No acute stops or thrombosis in the artery were seen. The aspiration pneumonia was seen on X-ray, there was not a CT scan of the brain made Table 3 .

**Table 3:** Overview of complications in CEA patients 19 21

	Clopidogrel ( *n* =80)		ASA-D ( *n* = 80)		*p* -Value
Cervical hemorrhage a	0 (0)	(95% CI 0.0–4.5)	1 (1.2)	(95% CI 0.03–6.7)	1.0
Ischemic stroke: ipsilateral	2 (2.5)	(95% CI 0.7–8.6)	1 (1.2)	(95% CI 0.03–6.7)	1.0
Ischemic stroke: contralateral	0 (0)	(95% CI 0.0–4.5)	0 (0)	(95% CI 0.0–4.5)	NC
Mortality (≤30 d)	2 (2.5)	(95% CI 0.7–8.6)	0 (0)	(95% CI 0.0–4.5)	0.50
Neuropraxia of cranial nerves	7 (8.8)	(95% CI 3.6–17.2)	9 (11)	(95% CI 5.3–20.3)	0.86
Persisting (≥30 d)	3 (42.8)		4 (44.4)		
Transient (< 30 d)	4 (57.2)		5 (55.5)		
Headache and/or hypertension	51 (63.8)	(95% CI 52.2–74.2)	61 (76.3)	(95% CI 65.4–85.1)	0.12
Persisting headache (≥30 d)	1 (12.5)		0 (0)		

Abbreviations: 95%CI, 95% confidence interval; ASA-D, acetylsalicylic acid and extended release dipyridamole; NC, not computable.

*Note* : Data are presented as *n* (%) unless stated otherwise.

a Requiring secondary surgery.

## Discussion

In this retrospective analysis no differences were found in peri- and postoperative stroke and hemorrhage after CEA between patients using clopidogrel monotherapy and ASA-D. There were no differences found in the other defined outcomes in both groups (clopidogrel monotherapy and ASA-D).

The limitation of this study is its retrospective nature which could imply inaccurately recording of some peri- and postoperative clinical parameters, e.g., hematoma. Therefore we interpret the results cautiously. We use these data as step up study for further research. Also the case-mix, treatment and outcome are impressible to a certain risk of bias. The incidence of cervical bleeding/hematoma after CEA is not easy to observe reliably and there could have been substantial difference in its definition. Nevertheless major bleeding, in need of a reoperation were well documented and therefore considered as an objective count. Postoperative there are clear symptoms for the nurse and doctor to check. If there was any suspicion of a stroke a neurologist was consulted. Therefore the primary endpoints can be assumed to be well documented.

There is a significant difference between the clopidogrel monotherapy and the ASA-D groups regarding the use of a patch. This may be true or could contain a risk for bias. A patch is used depending on the diameter of the carotid artery, a variable that was not reported in all operation reports. Some studies previously compared the effect of antiplatelet therapy as secondary prevention after stroke. 22 23 24 Studies specifically comparing the effect of antiplatelet therapy prior to and shortly after CEA are scarce. In the study by Stone et al (2011), reoperation for bleeding was significantly higher in the ASA/clopidogrel group (1.4%) versus ASA monotherapy (1.2%) or clopidogrel monotherapy (0.7%) after CEA (LoE 2b). 7 In the study by Weinrich et al (2014), the incidence of revision surgery was also higher for patients on ASA in combination with any other antiplatelet drug (commonly clopidogrel) when compared with ASA monotherapy (LoE 2b). 25 In both studies the incidence of postoperative CVA or TIA's are not mentioned. Other studies confirmed these findings. 26 27 When comparing ASA-D dual therapy to triple therapy (dipyridamole, ASA, and clopidogrel) no significant differences in postoperative microembolic signals or bleeding complications were demonstrated. 28

Literature suggests the costs of clopidogrel are lower than ASA-D. The costs of clopidogrel monotherapy in the Netherlands vary between €1.18 to 11.83 per patient/per 30 days depending on the manufacturer (with a maximum of €143.98 annually). The combination ASA-D costs at minimum €15.24 and €16.13 per patient/per 30 days at maximum (and a maximum of €196.29 annually). 29

The results of this study are in line with previously published literature. 30 The changed antiplatelet protocol did not result in more complications in our consecutive series. Nevertheless more clinical research may be needed to confirm or reject these data before firm conclusions can be drawn. It can be hypothesized that clopidogrel monotherapy could contribute to general therapy compliance and seems to be more cost effective on annual basis for the CEA patients in our investigated series. It may be suggested that clopidogrel should be the preferred choice for CEA patients.

## Conclusion

In this retrospective consecutive series the incidence of postoperative ischemic complications and perioperative hemorrhage after CEA seemed to be comparable in patients using clopidogrel monotherapy versus acetylsalicylic acid and extended release dipyridamole for secondary prevention after a cerebrovascular event. This study fuels the hypothesis that short- and midterm complications of clopidogrel and the combination acetylsalicylic acid and extended release dipyridamole are comparable.
